# Inflammation, Diabetes, and Chronic Kidney Disease: Role of Aerobic Capacity

**DOI:** 10.1155/2012/750286

**Published:** 2012-04-09

**Authors:** Flávio Gobbis Shiraishi, Fernanda Stringuetta Belik, Viviana Rugolo Oliveira e Silva, Luis Cuadrado Martin, João Carlos Hueb, Renato de Souza Gonçalves, Jacqueline Costa Teixeira Caramori, Pasqual Barreti, Roberto Jorge da Silva Franco

**Affiliations:** ^1^Division of Nephrology, Department of Internal Medicine, Botucatu Medical School, São Paulo State University (UNESP), 18618-000 Botucatu, SP, Brazil; ^2^Division of Cardiology, Department of Internal Medicine, Botucatu Medical School, São Paulo State University (UNESP), 18618-000 Botucatu, SP, Brazil

## Abstract

The persistent inflammatory state is common in diabetes and chronic kidney disease (CKD). These patients present exercise intolerance and increased arterial stiffness. Long-term aerobic exercise has been associated with better arterial compliance, antidiabetic and antiinflammatory benefits. We assessed the hypothesis that in patients with diabetes and CKD, better aerobic capacity is associated with less inflammatory state and arterial stiffness. Thirty-nine CKD patients (17 in hemodialysis) were evaluated. According to CKD etiology two patient groups were obtained: group of diabetics (GD) was formed by 11 patients and nondiabetics (GND) formed by 28 patients. Central blood pressure and arterial stiffness were evaluated by Sphygmocor device. Carotida intima-media thickness (CA-IMT) was evaluated by ultrasonography. Aerobic capacity was measured by estimated VO_2_max according to treadmill test by Bruce protocol. The GD showed a higher frequency of C-reactive protein above laboratory cutoff (*P* = 0.044), higher frequency of male gender, and a non significant higher value of VO_2_max (*P* = 0.099). The CA-IMT was similar. Only better aerobic capacity was associated with lower frequency of high C-reactive protein when adjusted to diabetes and gender in a logistic regression model. In conclusion, aerobic capacity was associated with inflammatory state, in CKD patients, independently of diabetes presence.

## 1. Introduction 

Chronic kidney disease (CKD), characterized by irreversible loss of renal function [1], is a major public health problem in the world. The prevalence of CKD increases with age and reaches around 17% of individuals over 60 years [2]. Cardiovascular (CV) disease is the leader cause of morbidity and mortality in these patients.

One of the major risk factors for development of CKD is diabetes, which causes, in addition to kidney damage, several cardiovascular comorbidities and visual and peripheral vascular complications. Persistent chronic microinflammatory state is very common in diabetic and CKD patients. It is associated with malnutrition and cardiovascular disease and is a potent predictor of mortality [3].

Increased arterial stiffness has been recognized as important predictors of CV diseases in patients with CKD and diabetes patients and is associated with chronic inflammatory state [4]. Like pulse wave velocity (PWV), central blood pressure (CBP), and augmentation index (AIx), carotid intima-media wall thickness (CA-IMT) is also a predictor of CV mortality in elderly patients with ischemic heart disease, hypertension, diabetes, and CKD [5].

CKD patients usually manifest symptoms of exercise intolerance such as muscle weakness and fatigue and are less active, exhibiting muscle atrophy even when compared to inactive normal subjects [6]. CKD patients substantially improve their strength, power, and muscle endurance, evaluated by exercise capacity and physical function tests after training [7]. Others have reported that training improve quality of life, maximum oxygen uptake (VO_2_max), muscle gain in mass and capillary density, velocity of nerve conduction, and blood pressure (BP) [8–11].

Recent evidence had shown that physical activity can ameliorate inflammatory state in CKD [12]. Two interventional studies showed a reduction of inflammatory markers with aerobic training [13, 14]. Another two studies, one interventional [15] and one transversal [16] have not demonstrated any effect in inflammatory state. Thus, the benefits of physical activity and its importance were recognized in diabetic patients from the general population, and physical training is fully recommended [17]. However, the effectiveness of physical activity in the prevention or improvement of inflammatory state in diabetic CKD patients had not yet been proved [15].

Therefore, the aim of this study is to evaluate the association between aerobic capacity and presence of diabetes, inflammation, arterial stiffness, and CA-IMT in chronic kidney disease patients.

## 2. Materials and Methods

Were included hemodialysis patients for at least six months or CKD predialysis patients. All subjects were aged more than 18 years. Subjects unable to perform exercise testing, with coronary heart disease or previous coronary artery bypass, active infection, cancer, or liver cirrhosis were excluded. Informed consent was obtained from each patient. This study was approved by Research Ethics Committee of the Botucatu Medical School with the protocol 3083/2009.

Clinical data evaluated were age, gender, body mass index, and kidney disease etiology. The following laboratorial data was evaluated: hemoglobin, calcium, phosphorus, parathyroid hormone, albumin, iron, ferritin, transferrin saturation, C-reactive protein, creatinine, and creatinine clearance. All laboratorial evaluations were performed in the morning while fasting. The hemodialysis patients were evaluated in the morning while fasting in the interdialytic day.

The C-reactive protein was evaluated by dry chemistry (VITROS kit, Ortho-Clinical Diagnostics, Rochester, NY, USA), and test sensitivity was 0.1 mg/dL. Elevated CRP was defined when a patient had values superior to 1 mg/dL, as defined by superior limit of our laboratory.

Patients who met the inclusion criteria underwent Sphygmocor CPV (AtCor Medical, Australia) evaluation for CBP, PWV, and AIx. The measurements were performed in supine position. The BP stabilization was considered when the difference between three consecutive measurements in five-minute interval was not greater than 5 mm Hg. Average and maximum of left and right CA-IMT by ultrasonography according to Mannheim carotid intima-media consensus [18] were evaluated.

For the estimation of VO_2_max, the patients were submitted to a treadmill test (Bruce protocol [19]), and the appropriate formula for nonathletes was used to calculate this variable as follows: VO_2_max = (time × 3.29) + 4.07 for men, and VO_2_max= (time × 3.36) + 1.06 for women. All evaluations were performed on the same day in the same sequence. Hemodialysis patients underwent their examinations on interdialytic days.

According to the kidney disease etiology, two patient groups were obtained. Group D (GD) formed by diabetic patients and group ND (GND) formed by nondiabetic patients. The groups were compared regarding age, body mass index, and laboratorial data, systolic, diastolic and pulse CBP, PWV, Aix, and CA-IMT.

For comparison between groups, *t*-test or Mann-Whitney was used when appropriate. Multiple logistic regressions were performed to identify variables independently associated with inflammation and selected variables with statistical probability of <0.1 between groups. Values were expressed as mean ± standard deviation to parametric variables or median (first–third quartile) when appropriate. A *P* < 0.05 was considered statistically significant.

## 3. Results

 There were included 39 patients (17 in hemodialysis). Clinical and laboratory characteristics are shown in Table 1. The GD was formed by 11 diabetic patients and the GND by 28 nondiabetic patients, there were 13 (46%) patients in hemodialysis in GND and four (36%) in GD (*P* = 0.725). Other clinical and laboratorial variables of the groups are shown in Tables 2 and 3, respectively. The number of male patients was higher in GD compared to GND, with *P* = 0.011.

Diabetic group had a CRP value of 1.50 (0.32–2.72) mg/dL and GND 0.5 (0.20–0.75) mg/dL. When patients were divided according to the inflammatory status (CRP > 1 mg/dL or CRP ≤ 1 mg/dL) a significantly greater number of chronic inflammatory state patients was found in GD (*P* = 0.044; Table 3). The values of creatinine, creatinine clearance, hemoglobin, calcium, phosphorus, parathyroid hormone, albumin, ferritin, and transferrin saturation were not different between groups (Table 3).

Was observed a trend of higher VO_2_max values in the patients without diabetes compared to diabetic patients (27.9 ± 8.09 mL/kg/min in GND and 23.3 ± 7.08 mL/kg/min in GD, *P* = 0.099; Table 2). Reanalyzing VO_2_max by gender, that is, excluding female sex from analysis, we have VO_2_max of 30.9 ± 8.58 mL/kg/min in nondiabetic group (*n* = 9 non-diabetic men) and 23.5 ± 7.44 mL/kg/min (*P* = 0.042) in diabetic group (*n* = 13 diabetic men), so, reinforcing the difference in VO_2_max between diabetics and nondiabetics. Thickness of the intimae-media right carotid artery showed a trend of higher values in diabetic patients (0.9 ± 0.29 mm in GD and 0.76 ± 0.216, *P* = 0.081), other indexes of carotid thickness were similar between groups. Comparing the values of arterial stiffness indexes, GD had similar values of GND.

**Figure 1:**
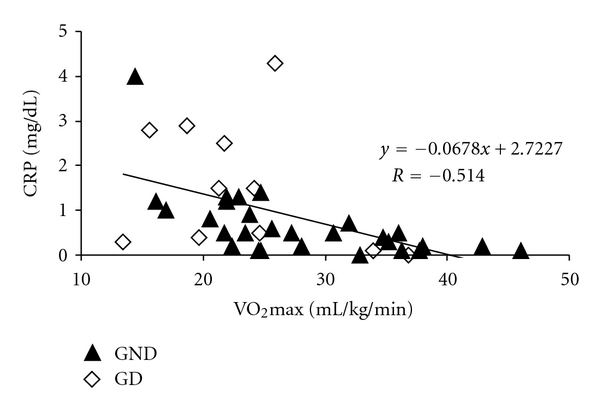
Correlation between C-reactive protein and VO_2_max. GND: nondiabetic group; GD: diabetic group.

In multiple logistic regression, with CRP as dependent variable (Table 4), the main determinant of C-reactive protein was VO_2_max (*P* = 0.024). Gender and diabetes were not independently associated with inflammatory state (*P* = 0.210 and *P* = 0.107, resp.).  Figure 1 shows the negative correlation between C-reactive protein and VO_2_max, in all patients, where the higher the aerobic capacity, the lower their inflammatory state (*R* = −0.514, *P* < 0.001).

## 4. Discussion

The C-reactive protein is a sensitive marker of inflammation, and an increase in its levels has been associated with higher risk of vascular disease [20]. Diabetic CKD patients' have high concentration of CRP, and improvement of aerobic capacity could affect that variable. The main result of this work is that CRP was not independently associated with diabetes when we take into account the VO_2_ level and that the VO_2_ level had a strong association with CRP independently of diabetes. We can speculate that it is possible that the inflammatory state of chronic kidney disease diabetic patients would be caused, in grand part or at least in part, by physical deconditioning. This speculative possibility must be confirmed in other studies with a great number of individuals and especially in longitudinal and interventional studies.

The long-term exercise training increases the nitric oxide availability and diminishes the oxidative stress, inflammation and blood pressure, which improves the endothelial function and consequently cardiovascular mortality [21–23]. Patients with coronary artery disease submitted to adequate chronic training with increase in the VO_2_max have lower levels of CRP compared with basal values [24]. Nitric oxide, a potent anti-inflammatory molecule, plays a role in this mechanism [24].

In the elderly, training also decreased the concentration of CRP [20, 25]. These data are consistent with our study, since individuals with higher aerobic capacity also had a lower inflammatory state. In diabetes, some recent studies have demonstrated the role of physical training in reducing CRP [26–29]. Experimental studies have also demonstrated the benefits of physical activity in reducing inflammatory markers and oxidative stress in animals with CKD [30, 31].

According to the American College of Sports Medicine and American Heart Association, to reduce risk of cardiovascular events, individuals with chronic illnesses should perform moderate-intensity physical activity, 30 min, 5 times weekly [32].

Physical capacity proved to be an important predictor of survival. In a study that evaluated patients with CKD and followed them over three years, patients with a VO_2_max superior to 17.5 mL/kg/min had a higher survival compared to individuals with lower peak VO_2_ [33]. The mean of VO_2_max of the current study was 26.6 ± 8.02 mL/kg/min.

Although the benefits of physical training have been demonstrated in CKD patients [11, 34–36], the results are controversially related to inflammatory markers, and few studies have demonstrated the effects of a physical activity program on CRP in these patients. A recent pilot study of obese diabetic CKD patients did not show any change in CRP after a period of aerobic training [15]. In the current study, a multiple logistic regression identified VO_2_max as a principal determinant of C-reactive protein. Our results were in accordance with previous studies [37–39] and reinforce the possibility that diabetic CKD patients would be benefited engaging in a physical activity program.

In the present study, measures of intima-media thickness of carotid arteries and arterial stiffness did not correlate with VO_2_max. So, better physical aerobic capacity was associated only with the metabolic marker of inflammation and not with structural or functional properties of arteries. However, these data do not exclude that long-term assisted physical training could have an impact on those parameters not observed in this transversal study.

In the current study, some limitations must be recognized. First, a small number of subjects was evaluated, nevertheless this number was sufficient to detect statistically significant correlations. This is a transversal study, so it submits to limitations inherent to this design and must be confirmed in a longitudinal and interventional study. However, we evaluated the confounding variables and took into account those in multiple analysis. Finally, the VO_2_max was indirectly measured.

In conclusion, better VO_2_max was associated with lower frequency of elevated CRP levels in subjects with chronic kidney disease. The current study strengthens previous findings and reinforces the hypothesis of cardiovascular benefits with a better aerobic capacity in CKD diabetic patients.

## Figures and Tables

**Table 1:** Clinical variables and laboratorial characteristics of patients.

Patients	39
Hemodialysis	17
Predialysis	22
Age (years)	54.9 ± 14.16
Kidney disease etiology	
Hypertension	16
Diabetes	11
Glomerulopathies	7
Others	5
BMI (Kg/m^2^)	26.5 ± 5.25
Central SBP (mm Hg)	122 ± 19.3
Central DBP (mm Hg)	83 ± 12.8
Central PP (mm Hg)	39 ± 13.4
Pulse wave velocity (m/s)	8.3 ± 1.26
Augmentation index (%)	26.3 ± 14.02
VO_2_max (mL/kg/min)	26.6 ± 8.02
CA-IMT L—m (mm)	0.79 ± 0.194
CA-IMT L—mx (mm)	0.96 ± 0.234
CA-IMT R—m (mm)	0.81 ± 0.247
CA-IMT R—mx (mm)	0.97 ± 0.273
Presence of atherosclerotic plaque (%)	41%
Creatinine (mg/dL)	6.0 ± 4.29
Clearance of creatinine (mL/min)	32 ± 20.4
Hemoglobin (g/dL)	12 ± 1.7
Calcium (mg/dL)	9.3 ± 0.79
Phosphorus (mg/dL)	4.8 ± 1.48
CRP (mg/dL)	0.9 ± 1.06
PTH (pg/mL)	493 ± 553.3
Albumin (g/dL)	4 ± 0.45
Ferritin (g/dL)	635 ± 568.1
Transferrin saturation (%)	33.5 ± 15.77

BMI: body mass index; SBP: systolic blood pressure; DBP: diastolic blood pressure; PP: pulse pressure; PWV: pulse wave velocity; AIx: augmentation index; CA-IMT L—m: mean of left carotid intima-media thickness; CA-IMT L—mx: maximal of left carotid intima-media thickness; CA-IMT R—m: mean of right carotid intima-media thickness; CA-IMT R—mx: maximal of right carotid intima-media thickness; CRP: C-reactive protein; PTH: parathyroid hormone.

**Table 2:** Clinical Variables between groups.

	Diabetics (*n* = 11)	Nondiabetics (*n* = 28)	*P*
Age (years)	60 ± 13.7	53 ± 13.9	0.126
Gender: male/female	10 M/1 F	13 M/15 F	0.011
BMI (Kg/m^2^)	28 ± 3.4	26 ± 5.8	0.298
Central SBP (mm Hg)	117 ± 19.5	124 ± 19.3	0.344
Central DBP (mm Hg)	74 ± 10.4	87 ± 12.1	0.005
Central PP (mm Hg)	43 ± 15.1	37 ± 12.5	0.205
PWV (m/seg)	8.3 ± 1.36	8.3 ± 1.24	0.884
AIx (%)	21 ± 14.1	28 ± 13.6	0.124
VO_2_max (mL/kg/min)	23.3 ± 7.08	27.9 ± 8.09	0.099
CA-IMT L—m (mm)	0.8 ± 0.17	0.78 ± 0.202	0.328
CA-IMT L—mx (mm)	1.0 ± 0.20	0.94 ± 0.248	0.459
CA-IMT R—m (mm)	0.9 ± 0.29	0.76 ± 0.216	0.081
CA-IMT R—mx (mm)	1.1 ± 0.28	0.92 ± 0.258	0.112
Presence of atherosclerotic plaque (%)	54.5	35.7	0.383

BMI: body mass index; SBP: systolic blood pressure; DBP: diastolic blood pressure; PP: pulse pressure; PWV: pulse wave velocity; AIx: augmentation index; CA-IMT L—m: mean of left carotid intima-media thickness; CA-IMT L—mx: maximal of left carotid intima-media thickness; CA-IMT R—m: mean of right carotid intima-media thickness; CA-IMT R—mx: maximal of right carotid intima-media thickness.

**Table 3:** Laboratorial data.

	Diabetics (*n* = 11)	Nondiabetics (*n* = 28)	*P*
Creatinine (mg/dL)	5.8 ± 4.14	6.1 ± 4.42	0.863
Creatinine clearance (mL/min)	23 ± 9.6	36 ± 22.8	0.206
Hemoglobin (g/dL)	11.4 ± 1.51	12.3 ± 1.71	0.159
Calcium (mg/dL)	8.9 ± 0.69	9.4 ± 0.80	0.109
Phosphorus (mg/dL)	4.9 ± 1.40	4.7 ± 1.52	0.633
CRP (mg/dL)	1.50 (0.32–2.72)	0.5 (0.20–0.75)	0.021
CRP > 1.0 mg/dL	6	6	0.044
CRP ≤ 1.0 mg/dL	5	22	
PTH (pg/mL)	454 ± 473.2	509 ± 591.9	0,787
Albumin (g/dL)	3.9 ± 0.46	4.0 ± 0.44	0.465
Ferritin (g/dL)	740 ± 792.2	571 ± 388.7	0.448
Transferrin saturation (%)	40 ± 13.4	30 ± 16.23	0.142

CRP: C-reactive protein; PTH: parathyroid hormone.

**Table 4:** Multiple linear regression: C-reactive protein as dependent variable.

	*P*	RR	95.0% C.I.	
			Lower	Upper
Gender	0.210	0.221	0.021	2.335
Diabetes	0.107	7.487	0.646	86.803
VO_2_max (mL/Kg/min)	0.024	0.827	0.701	0.976
